# Angling for Thyroid Answers: Study Links PBDEs to Hormone Disruption in Male Sport-Fish Consumers

**Published:** 2008-12

**Authors:** Richard Dahl

Levels of polybrominated diphenyl ethers (PBDEs) measured in human samples have increased in recent years, but the health effects of these compounds are not well studied. A group of persistent pollutants similar in structure to polychlorinated biphenyls (PCBs), PBDEs are thought to affect endocrine function, but this relationship has only been examined in several small studies. A new study significantly expands this knowledge base by analyzing PBDE exposure among a large cohort of male sport-fish consumers and concluding that these exposures are associated with increased thyroglobulin antibodies and increased thyroxine (T_4_) in adult males independent of PCB exposure **[*EHP*** **116:1635–1641; Turyk et al.]**.

The study examined 405 adult males who consumed sport fish from the Great Lakes during the early 1990s. Researchers gathered data on the subjects’ levels of fish consumption, medical diseases, and use of medications, and took serum samples that were tested for PBDEs, PCBs, and DDE, a metabolite of DDT that may affect thyroid hormones. Total and free T_4_ and triiodothyronine (T_3_) were measured in serum and urine.

PBDE concentrations were positively associated with increased T_4_ and reverse T_3_, and inversely correlated with total T_3_ and thyroid-stimulating hormone (TSH). In addition, PBDEs were positively related to the percentage of T_4_ bound to albumin, a carrier protein. An observed increase in thyroglobulin antibodies in men with the highest PBDE exposures may indicate an increased susceptibility to autoimmune thyroiditis among people who have been exposed to PBDEs, according to the authors.

The findings of a positive association of PBDEs with T_4_ are not consistent with results of animal studies that have shown decreased T_4_ in rats and mice exposed to PBDE. However, the results do align with those of several smaller human studies. The authors speculate the disparity may be attributable to the fact that, while thyroid hormone regulation is similar among vertebrates, some functions differ by species.

A major strength of the study is the measurement of the effects of PBDEs on multiple hormones and the consideration of other environmental exposures that can affect thyroid hormones. The authors point out that their findings provide a rationale for future mechanistic studies related to PBDE exposure, including how those exposures may be linked to changes in thyroid hormone metabolism and binding of T_4_ to serum-binding proteins. Also needed, they write, are larger studies to determine whether PBDE exposure is related to thyroid disease in human populations.

## Figures and Tables

**Figure:**
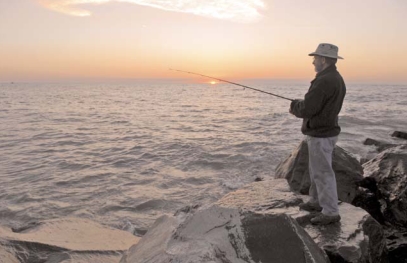
EPA data show that contaminant levels in fish from Lake Michigan (above) and the other Great Lakes, while still high, have declined consistently since 1990.

